# The association of sex-biased ATRX mutation in female gastric cancer patients with enhanced immunotherapy-related anticancer immunity

**DOI:** 10.1186/s12885-021-07978-3

**Published:** 2021-03-07

**Authors:** You Ge, Feiran Wei, Guoping Du, Gaoqiang Fei, Wei Li, Xiaoshan Li, Jinjin Chu, Pingmin Wei

**Affiliations:** 1 Department of Epidemiology and Health Statistics, School of Public Health, Southeast University, 87 Dingjiaqiao Road, Nanjing, 210009 Jiangsu China; 2 Department of Hematology and Oncology, Zhongda Hospital, School of Medicine, Southeast University, Nanjing, China; 3 Southeast University Hospital, Nanjing, China; 4 Department of Lung Transplant Center, The Affiliated Wuxi People’s Hospital of Nanjing Medical University, Wuxi, Jiangsu China

**Keywords:** Gastric cancer, Sex biases, ATRX mutation, Anticancer immunity

## Abstract

**Background:**

Genetic alterations have been proven to be the promising biomarkers for ICI response. However, sex biases in genetic alterations have been often ignored in the field of immunotherapy, which might specially influence the anticancer immunity and immunotherapy efficacy in male or female patients. Here, we have systematically evaluated the effect of the sex biases in somatic mutation of gastric cancer (GC) patients on the anticancer immunity and clinical benefit to immunotherapy.

**Methods:**

Genomic and transcriptomic data of gastric cancer were downloaded from The Cancer Genome Atlas (TCGA) and International Cancer Genome Consortium (ICGC). We also obtained the genomic and clinical data of a MSKCC ICI-treated cohort from cbioportal database. GC male and female-derived tumor somatic mutation profiles were compared by maftools R package. Single sample gene set enrichment analysis (ssGSEA) was conducted to calculate the score of the anticancer immunity indicators including IFN-γ signaling, cytolytic activity (CYT) and antigen presenting machinery (APM).

**Results:**

ATRX was found to mutate more frequently in female GC patients compared to male patients (FDR = 0.0108). Female GC patients with ATRX mutation manifested significantly more MSI-high subtypes, increased TMB and PDL1 expression as well as higher scores of IFN-γ signaling, CYT and APM. Gene set enrichment analysis (GSEA) has shown that ATRX mutation might enhance the immunogenicity and anticancer immunity through affecting DNA damage repair pathways. In the ICI-treated cohort from MSKCC, GC patients with ATRX mutation were associated with prolonged overall survival. When stratifying the entire ICI-treated cohort by sex, female patients with ATRX mutation obtained significantly better survival benefits than that of ATRX mutant male patients (Female patients, HR of ATRX MT vs WT = 0.636, 95%CI = 0.455–0.890, *P* = 0.023; Male patients, HR of ATRX MT vs WT = 0.929, 95%CI = 0.596–1.362, *P* = 0.712).

**Conclusions:**

ATRX mutation might serve as a potential predictive biomarker for favorable clinical benefit to ICI in female GC patients. ATRX mutation could be applied in combination with other biomarkers of ICI response to better identify the female GC patients who will derive greater benefits from ICI therapy.

**Supplementary Information:**

The online version contains supplementary material available at 10.1186/s12885-021-07978-3.

## Background

Gastric cancer (GC) is a major global health problem. In China, GC is the second most common type of tumors and account for 291,000 attributed fatalities in 2015 [1]. The efficacy of current treatment options for advanced GC is limited, and the overall survival of these patients is still poor. The development of tumor immunotherapy, represented by immune checkpoint inhibitors (ICI), has reshaped the treatment of solid tumors and proven efficacy in some malignancy, such as melanoma, non-small cell lung cancer, renal cell carcinoma and recurrent squamous cell carcinoma of the head and neck [2–5]. Although ICIs have shown encouraging preliminary efficacy in advanced GC, the overall response to ICI might not be as remarkable as to those solid tumors such as melanoma and non-small cell lung cancer [6]. Thus, further GC researches on development of accurate biomarkers that can predict the response to ICI are mandatory.

Specific somatic mutations, such as TET1 mutation, NOTCH mutation as well as TP53 and KRAS co-mutation, have been proven to be the promising biomarkers for ICI response [7–9]. However, sex biases, often ignored in the in the field of immunotherapy, might exist in the specific genetic alterations. By making a comparison on somatic mutation profiles between tumors occurring in men and in women across numerous cancer types, Li et al. have discovered that there existed large sex biases in mutation density and frequency, which would influence the prognostic biomarker performance [10]. Moreover, sex could also affect the efficacy of immunotherapy. For example, Wu et al. have reported that treatment with ICI prolongs the overall survival and progression-free survival of male patients with tumors, in particular those treated with CTLA-4 inhibitor [11]. Conforti et al. carried out two meta-analyses respectively and found male patients could derive a larger benefit from ICI alone than female patients, while women obtain more clinical benefits than men from ICI plus chemotherapy [12, 13]. These evidence provides a hypothesis that there might be certain sex-biased biomarkers more appropriate for male or female patients.

Therefore, we attempted to explore whether there are specific gene mutations with sex biases that would influence the efficacy of immunotherapy for female or male GC patients. In this study, we systematically evaluated the sex variance in GC somatic mutation profiles and discovered that ATRX mutation more frequently occurring in female GC patients was associated with higher TMB, increased anticancer immunity and favorable clinical benefit to ICI.

## Methods

### Data sources

We downloaded the level 3 RNA-seq data (*n* = 375), the corresponding clinical data (*n* = 443) and the somatic mutation profile (*n* = 433) of GC patients from TCGA. The patients without gene expression profile, sex information or mutation data were excluded. The somatic mutation profile of Chinese and Japanese GC patients (Project: GACA-CN, *n* = 123; GACA-JP; *n* = 585) was also downloaded from ICGC datasets. The MSI status data of GC patients from TCGA were obtained in the previous study [14]. The genomic and survival data of a ICI-treated cohort were downloaded from Samstein et al. [15].

### Somatic mutation analysis

The somatic mutation data were analyzed by maftools R package [16]. The mutation profiles between male and female GC patients were compared using the function of mafComapre. We only tested the genes mutated in at least in 5 samples in one of the group. The false discovery rate (FDR) of 0.05 was considered as the cut-off value for significance.

PolyPhen-2 (Polymorphism Phenotyping v2) is a software tool which can estimate the probability of protein structure damage caused by the missense mutation [17]. We used the Polyphen-2 software to further evaluate the mutational impact on protein structure. Based on the final score assessing influence of missense mutation on protein structure, the threshold value as 0.69 was set to divide the missense mutation into benign missense mutation or deleterious missense mutation.

### Tumor mutation burden (TMB) quantification

The somatic called variants determined by TCGA and ICGC were used to estimate the TMB. The size of the exome region was defined as 38 Mb. The synonymous mutation and variants in the intergenic or noncoding regions were excluded in the estimation of TMB. As outlined in the previous study [18], TMB score of each sample was calculated as the total number of mutations counted divided by exome region size.

### The estimation of cytolytic activity (CYT) score

The gene expression data in FPKM format collected from TGCA were first transferred to transcripts per million (TPM). The TPM values were then log2 transformed. CYT score was described as geometric mean of GZMA and PRF1 expression values in TPM [19].

### Evaluation of tumor infiltrating immune cells with CIBERSORT

CIBERSORT is a deconvolution tool that can accurately estimate the abundances of human leukocyte subsets in a tumor biopsy [20]. We used CIBERSORT method and the LM22 gene signature (a validated leukocyte gene signature matrix containing 22 functionally defined human hematopoietic subsets) to evaluate the proportions of immune cells in GC. At the criterion of *P* < 0.05, the results of the immune cell composition from CIBERSORT were considered to be accurate.

### Single sample gene set enrichment analysis (ssGSEA)

The method of ssGSEA computes a gene set enrichment score per sample by comparing the ranks of the genes in the gene list with the ranks of all other genes in the transcriptome [21]. We employed this approach to computationally assess the IFN-γ signaling and antigen presenting machinery (APM) [22, 23]. We have used a previously defined 7 genes APM signature that consisted of MHC class I genes and genes involved in processing and loading antigens. IFN-γ signaling was scored using the gene set from two clinical trials of advanced GC from KEYNOTE-012 [6] and KEYNOTE-059 [24]. The method of ssGSEA was implemented in GSVA R package [25]. The gene list was shown in Additional file 1.

### Gene set enrichment analysis

Gene set enrichment analysis (GSEA) was implemented by javaGSEA application (version 4.0.3). GSEA was used to identify the key pathways associated with the ATRX mutation status. The annotated gene sets (c2.cp.kegg.v7.1.symbols.gmt) for GSEA were selected as the reference gene sets. FDR < 0.25 and *P* < 0.05 were considered as the threshold criteria.

### Statistical analysis

Log-rank test in Kaplan-Meier analysis was performed to evaluate survival difference between groups. Wilcox test or Kruskal-Wallis test with post hoc pairwise Bonferroni correction was used to assess the differences in subgroups. Chi-square test or Fisher exact test was applied to compare the distribution of clinical features between groups. Univariate and multivariate Cox regression analyses were performed to identify the factors with independent prognostic value. *P* < 0.05 were chosen as the cut-off criteria.

All statistical analyses were carried out with R software (version 3.6.2). The figures of boxplot and histogram were produced by GraphPad Prism (version 8.0.1).

## Results

### Somatic variations in male and female GC patients

**Fig. 1:**
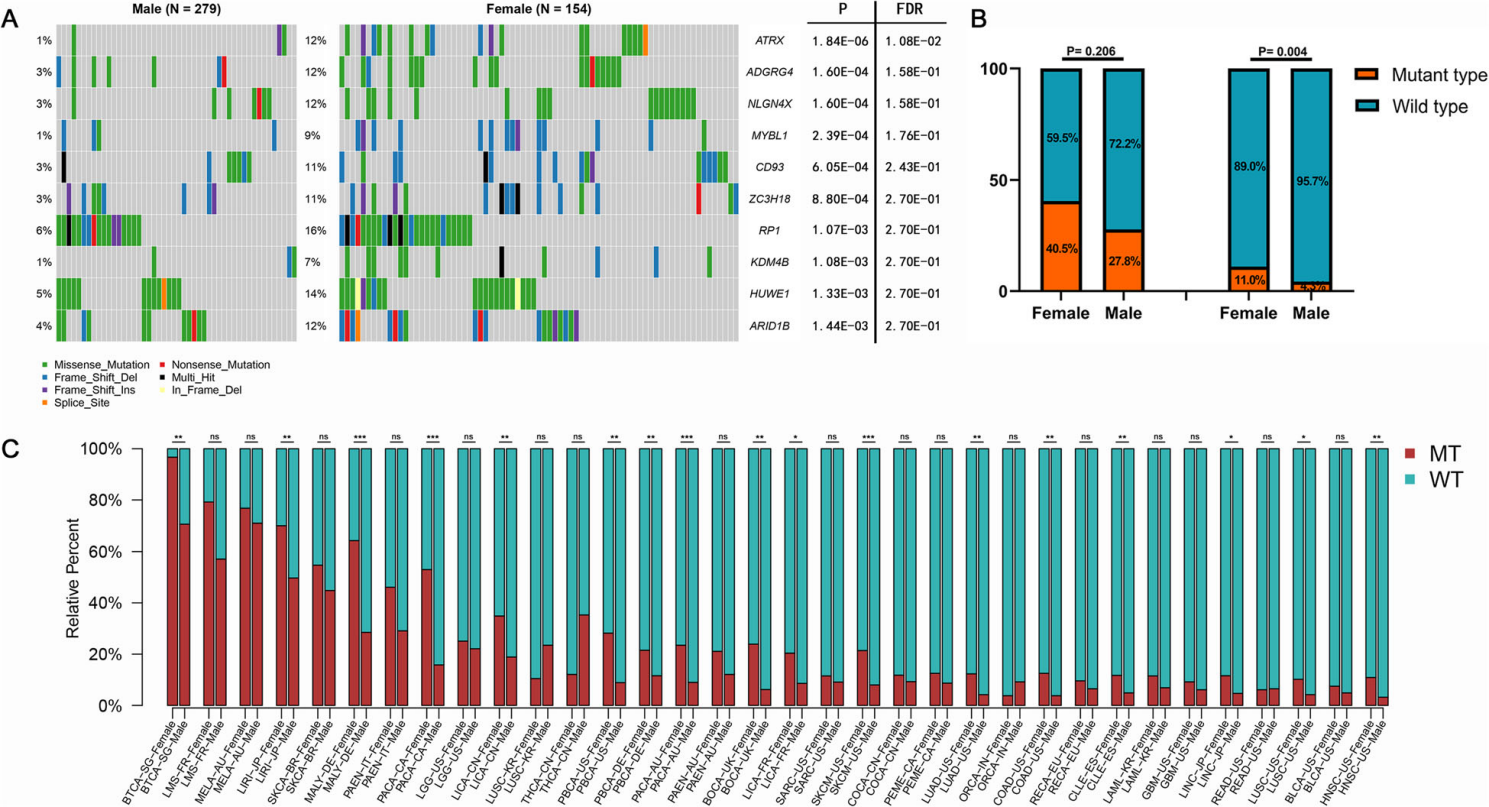
The comparison of ATRX mutation frequency between female and male patients. **a** Comparison of somatic mutation profiles between female and male GC patients. ATRX is the only gene found to significantly occur more in female patients compared to male patients (FDR = 0.0108). **b** Proportion of ATRX mutation in female and male GC patients in cohort of GACA-CN and GACA-JP. **c** Comparison of ATRX mutation frequency between female and male patients with various tumor types from ICGC database. ***, *P* < 0.001; **, *P* < 0.01; *, *P* < 0.05

We first compared the frequency of somatic mutations from TCGA cohort between female and male GC patients. Intriguingly, only ATRX mutation was found to significantly occur more in female patients compared to male patients after adjusting the false discovery rate (FDR = 0.0108) (Fig. 1a).

To avoid accidental bias caused by a single cohort, we further verified the sex biases of ATRX mutation in cohorts of GACA-CN and GACA-JP. The mutation frequency of ATRX in GACA-CN and GACA-JP was 31.7% (39/123) and 6.3% (37/585) respectively. The proportions of female patients with ATRX mutation in GACA-CN (female 40.5% vs male 27.9%, Fishers exact test, *P* = 0.206) and GACA-JP (female 11.0% vs male 4.3%, Fishers exact test, *P* = 0.004) were both higher than that of male patients (Fig. 1b). Subsequently, we attempted to examine whether the sex dipartites in ATRX mutation could be found in other cancer projects from ICGC. We excluded the sex-restricted tumors and cancer projects with ATRX mutation frequency less than 5%. Remarkably, half of cancer projects (50%, 17/34) showed significantly sex differences in ATRX mutation (Fig. 1c, Additional file 2). In most cancer projects (88.2%, 30/34) the mutation frequency of ATRX was higher in female patients than that of male patients, although the difference in some cancer projects did not show statistical significance. In addition, we evaluated the potential impact of ATRX missense mutation on protein structure by PolyPhen-2 software. The deleterious missense mutations in female patients were predominant among all kinds of ATRX mutation in TGCA cohort (Additional file 3). Overall, these results indicate the ATRX mutation in gastric cancer patients might exist sex biases.

### Correlation between ATRX mutation and clinical features

**Fig. 2:**
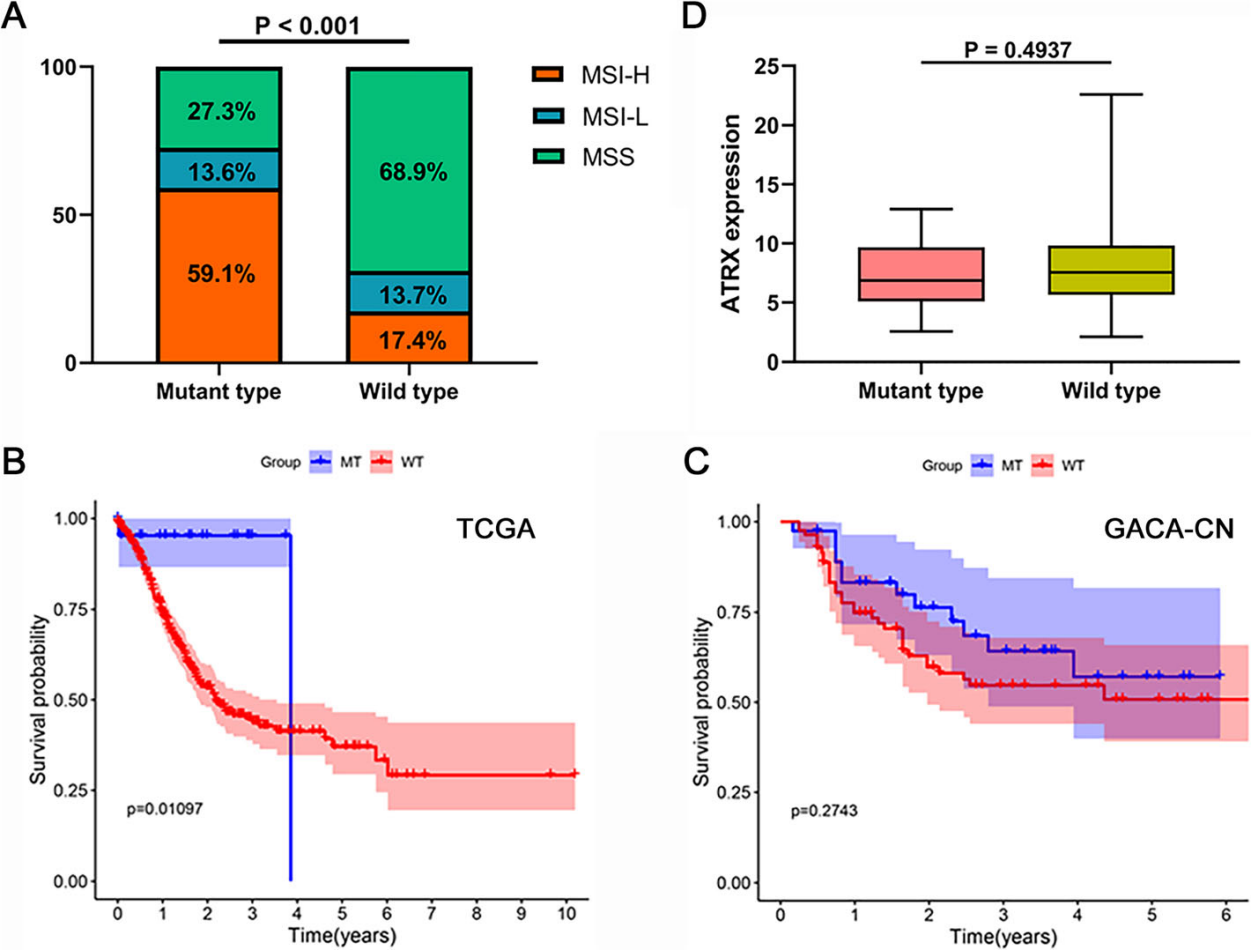
Correlation between ATRX mutation and clinical features. **a** Comparison of the proportion of MSI status between ATRX mutant and wild type GC patients. **b-c** Survival curves of overall survival in GC patients with or without ATRX mutation from cohorts of TCGA and GACA-CN. **d** The effect of ATRX mutation on ATRX expression

We first compared the distribution of clinical features between ATRX mutant type and wild type patients (Table 1). Remarkably, the majority of ATRX mutant patients were microsatellite instability-high (MSI-H) tumors compared to the ATRX wild type patients (Fig. 2a). Then we proceeded to examine whether ATRX mutation could affect the overall survival of GC patients. Log-rank test in Kaplan-Meier analysis demonstrated that ATRX mutant patients trended towards better overall survival than wild patients (Fig. 2b). Kaplan-Meier analysis of patients from GACA-CN also showed the similar result (Fig. 2c). Moreover, in order to better evaluate the survival benefits from ATRX mutation, we further explored the impact of ATRX mutation on prognosis outcomes in non-GC cohorts in TCGA database. We have selected the non-gastric cancer cohorts with ATRX mutation frequency more than 10%, including LGG (39.2%), UCEC (24.3%), SARC (16.9%), GBM (10.4%), CESC (10.4%), COAD (10.3%). Similarly, we found that ATRX mutation in patients with UCEC was also associated with prolonged overall survival (Additional file 4). Finally, we evaluated the effect ATRX mutation on ATRX expression. We found that ATRX mutant tumors had lower ATRX expression, although the difference did not manifest statistical significance (Fig. 2d). In summary, ATRX mutation was significantly correlated to MHS-H subtype and better over survival in GC patients.

**Table 1:** Distribution of clinical features between ATRX mutant and wild patients

Characteristics	Classification	ATRX mutant type	ATRX wild type	χ^2^	*P* value
MSI status	MSI-H	13	62	19.317	**< 0.001**
	MSS	6	246		
	MSI-L	3	49		
Age	≤ 65	8	181	0.867	0.325
	> 65	15	224		
Sex	Female	19	135	23.475	**< 0.001**
	Male	4	275		
Grade	G1	0	12	10.564	**0.010**
	G2	2	156		
	G3	20	234		
	GX	1	8		
Stage	Stage I	4	52	2.045	0.724
	Stage II	9	120		
	Stage III	7	172		
	Stage IV	2	41		
	Unknown	1	25		
T	T1	0	23	4.301	0.314
	T2	5	85		
	T3	15	180		
	T4	3	113		
	TX	0	9		
M	M0	21	360	0.902	1.000
	M1	1	29		
	MX	1	21		
N	N0	10	118	10.024	**0.029**
	N1	5	112		
	N2	7	76		
	N3	0	86		
	NX	1	16		

*MSI-H* Microsatellite instability-high, *MSS* Microsatellite stability, *MSI-L* Microsatellite instability-low

### ATRX mutation is associated with increased tumor mutation burden and higher expression of PDL1

**Fig. 3:**
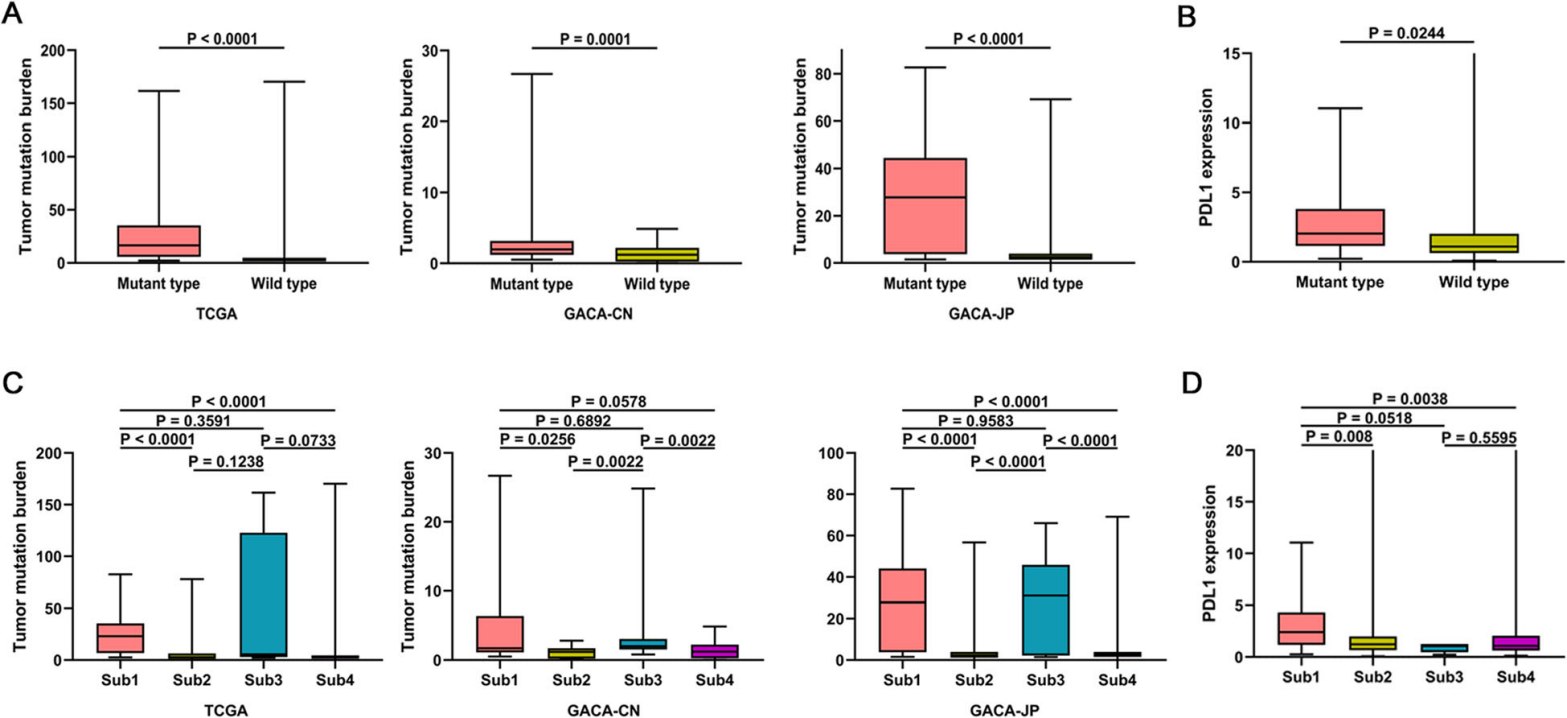
ATRX mutation is associated with increased tumor mutation burden and PDL1 expression. **a** Boxplot of TMB between ATRX mutant and wild type GC patients in cohorts of TCGA, GACA-CN and GACA-JP. **b** Elevated expression of PDL1 was observed in patients with ATRX mutation. **c-d** Correlation of ATRX mutation with TMB and PDL1 expression stratified by sex (Sub1: female patients with ATRX mutation; Sub2: female patients without ATRX mutation; Sub3: male patients with ATRX mutation; Sub4: male patients without ATRX mutation)

Tumor mutation burden (TMB) and PDL1 expression were two common indicators of predicting response to immunotherapy. We calculated the TMB of each GC patient from TCGA, GACA-CN and GACA-JP respectively. As shown in Fig. 3a, higher TMB was observed in ATRX mutant GC patients from cohort of TCGA (median TMB 16.61 vs 2.526, *P* < 0.0001), GACA-CN (median TMB 1.974 vs 1.211, *P* = 0.0001) and GACA-JP (median TMB 27.82 vs 2.421, *P* < 0.0001). We then compared the expression of PDL1 between GC patients with or without ATRX mutation in TCGA cohort. ATRX mutant patients harbored higher expression of PDL1 (median expression 2.027 vs 1.098, *P* = 0.0244) than that of wild patients (Fig. 3b).

Given the potential sex differences in ATRX mutation, we further to explore the correlation of ATRX mutation with TMB and PDL1 expression stratified by sex (Sub1: female patients with ATRX mutation; Sub2: female patients without ATRX mutation; Sub3: male patients with ATRX mutation; Sub4: male patients without ATRX mutation). In cohorts of GACA-CN and GACA-JP, both female and male patients with ATRX mutation had higher TMB than ATRX wild type patients (Fig. 3c). However, in cohorts of TCGA, only ATRX mutant type female patients showed higher TMB than other patients without ATRX mutation. There is no significant difference of TMB between male patients with or without ATRX mutation. Furthermore, in three cohorts no significant differences were observed between female and male patients with ATRX mutation. When comparing the PDL1 expression between the four subgroups in TCGA cohort, we found that the expression of PDL1 in ATRX mutant female patients was higher than patients without ATRX mutation (Fig. 3d).

In general, these results suggested that patients with ATRX mutation, especially female GC patients, were significantly associated with higher TMB and PDL1 expression.

### Female GC patients with ATRX mutation show enhanced anticancer immunity

**Fig. 4:**
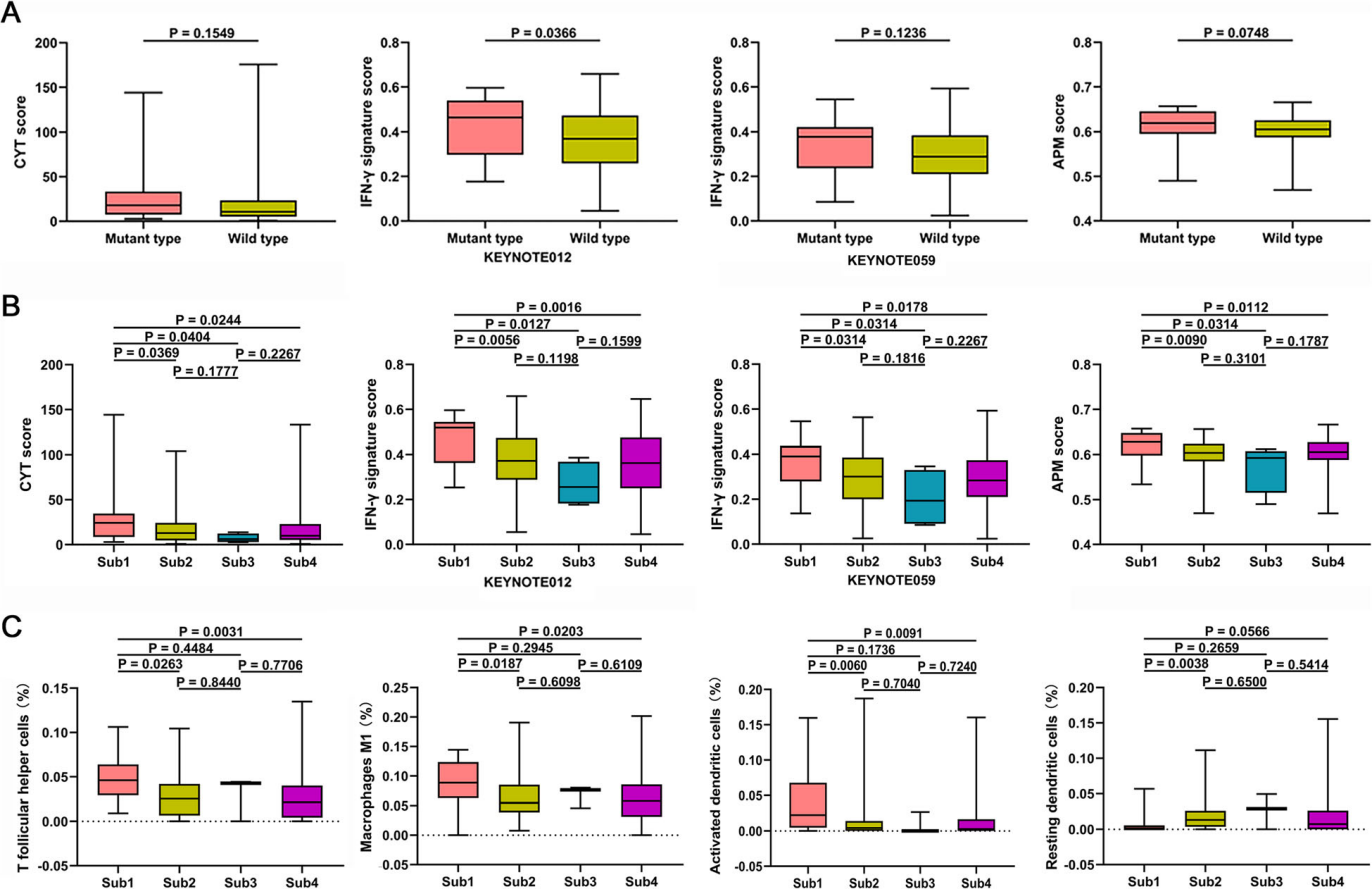
Female GC patients with ATRX mutation show enhanced anticancer immunity. **a** Boxplot of anticancer immunity indicators scores between ATRX mutant and wild patients. Although the scores of anticancer immunity in patients with ATRX mutation were higher than that in ATRX wild patients, the difference exhibited no statistically significant. **b** Comparison of the influence of ATRX mutation on anticancer immunity stratified by sex. Female patients with ATRX mutation show significantly higher scores than other patients. **c** Relative abundance fractions of immune cell population in four subgroups (Sub1: female patients with ATRX mutation; Sub2: female patients without ATRX mutation; Sub3: male patients with ATRX mutation; Sub4: male patients without ATRX mutation)

We resorted to several proven immunotherapy-related factors, including cytolytic activity (CYT) [26], IFN-γ signaling [6], antigen presenting machinery (APM) [27] and tumor infiltrating immune cells [28], to characterize the anticancer immunity. We have used the method of ssGSEA to explore the association of ATRX mutation with anticancer immunity. Although the scores of anticancer immunity in patients with ATRX mutation were higher than that in patients without ATRX mutation, the difference exhibited no statistically significant (Fig. 4a).

However, when assessing the influence of ATRX mutation on anticancer immunity stratified by sex, we found that female patients with ATRX mutation showed significantly higher scores of the immunotherapy-related factors than patients without ATRX mutation (Fig. 4b). There were no significant differences of anticancer immunity scores between ATRX mutant and wild male patients. Furthermore, female patients with ATRX mutation manifested higher scores of immunotherapy-related indicators than ATRX mutant male patients.

Finally, we used CIBERSORT method to investigate the proportions of infiltrating immune cells among ATRX mutant female patients (Sub1), ATRX wild female patients (Sub2), ATRX mutant male patients (Sub3) and ATRX wild male patients (Sub4). T follicular helper cells, M1 macrophages and activated dendritic cells were significantly enriched in female patients harboring ATRX mutation, while the proportions of resting dendritic cells were significantly lower than other patients (Fig. 4c). T follicular helper cells, M1 macrophages and activated dendritic cells are associated with antigen presentation machinery, which was consistent with the increased APM in female patients with ATRX mutation.

In conclusion, female GC patients with ATRX mutation showed stronger anticancer immunity than ATRX wild patients or mutant male patients. The enrichment of T follicular helper cells, M1 macrophages and activated dendritic cells in ATRX mutant female patients also supported the above result.

### The effect of ATRX mutation on anticancer immunity in female patients is not affected by sex-based immune heterogeneity

In order to examine whether the effect of ATRX mutation on anticancer immunity in female patients was due to the sex-based immune heterogeneity, we further compared the above anticancer immunity factors between male and female patients. Only TMB displayed the sex differences, and the female patients had higher TMB than male patients (Additional file 5**)**. However, the differences of TMB between women and men disappeared when we exclude the female samples with ATRX mutation (Additional file 5). These results revealed that the enhanced anticancer immunity in ATRX mutant female patients was not affected by sex-based immune heterogeneity.

### Independent prognostic analysis of ATRX mutation

**Fig. 5:**
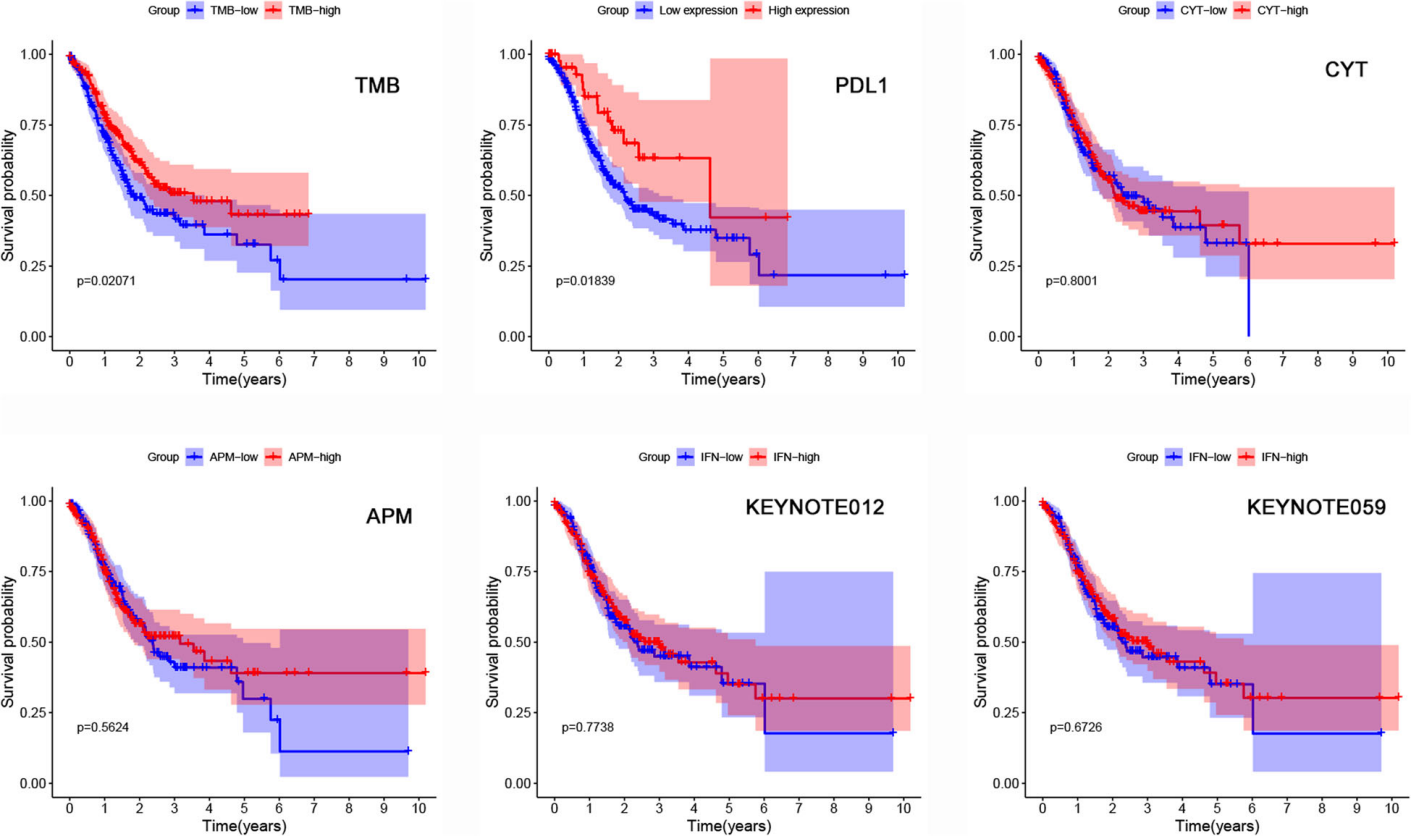
Prognosis value of TMB, PDL1 expression, CYT score, IFN-γ signaling score and APM score

We first carried out the Kaplan-Meier analysis to determine the prognostic values of the above immunotherapy-related factors. The TMB and PDL1 expression were divided into high and low groups on the basis of the X-tile tool. As shown in Fig. 5, the TMB and PDL1 expression exerted the influence on patients’ survival. The high group of TMB and PDL1 implied a better outcome. We then included TMB and PDL1 expression into the subsequent analysis to examine the independent prognostic value of ATRX mutation. Univariate and multivariate Cox regression analyses illustrated that ATRX mutation was the independent prognostic factor (Table 2).

**Table 2:** Univariate and multivariate Cox regression analysis of clinical pathologic features

Characteristics	Classification	Univariate analysis			Multivariate analysis		
		HR	95%CI	*P* value	HR	95%CI	*P* value
Age	< 60						
	> = 60	1.734	1.128–2.667	**0.012**	2.021	1.310–3.118	**0.001**
Sex	female						
	male	1.263	0.844–1.889	0.256			
ATRX	wild type						
	mutant type	0.122	0.017–0.875	**0.036**	0.132	0.018–0.947	**0.044**
Grade	grade1			0.270			
	grade2	1.159	0.159–8.445	0.884			
	grade3	1.581	0.220–11.383	0.649			
Stage	Stage I			**0.002**			**0.001**
	Stage II	1.413	0.642–3.108	0.390	1.561	0.708–3.444	0.270
	Stage III	2.333	1.117–4.873	**0.024**	2.519	1.204–5.270	**0.014**
	Stage IV	3.634	1.578–8.370	**0.002**	4.334	1.875–10.017	**0.001**
MSI status	MSS			0.116			
	MSI-L	1.326	0.795–2.212	0.280			
	MSI-H	0.659	0.467–1.111	0.118			
TMB	Low						
	High	0.625	0.433–0.904	**0.012**	0.738	0.504–1.079	0.116
PDL1	Low						
	High	0.427	0.208–0.878	**0.021**	0.464	0.225–0.956	**0.037**

*MSI-H* Microsatellite instability-high, *MSS* Microsatellite stability, *MSI-L* Microsatellite instability-low

### Impact of ATRX mutation on DNA damage repair

**Fig. 6:**
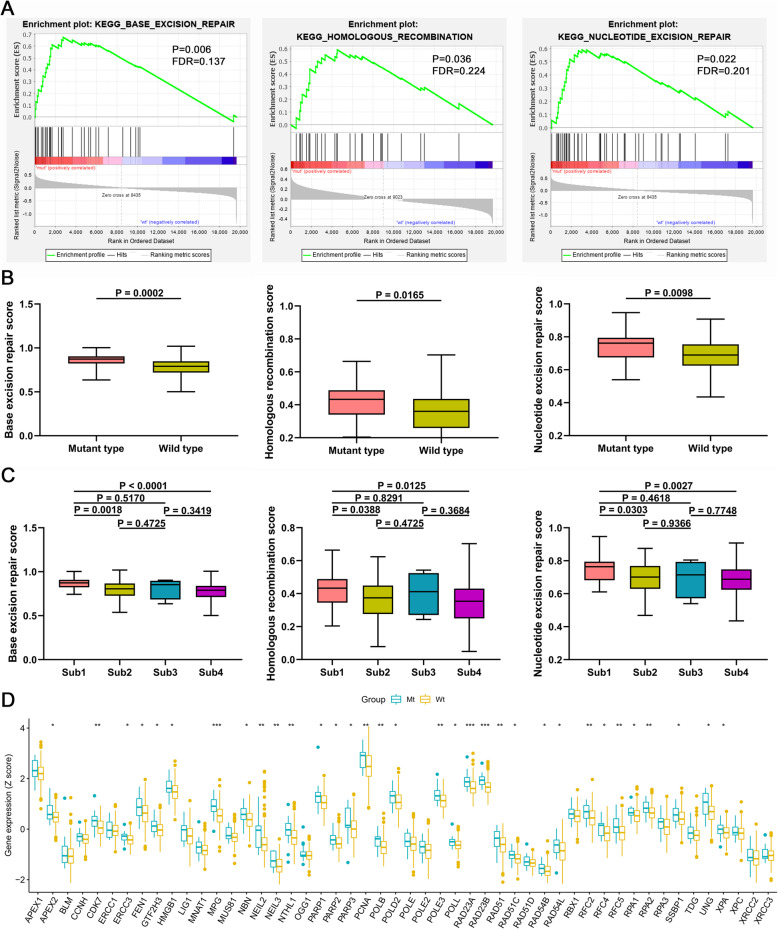
Impact of ATRX mutation on DNA damage repair. **a** GSEA analysis showed that the pathways of base excision repair (BER), nucleotide excision repair (NER) and homologous recombination repair (HRR) were significantly enriched in ATRX mutant patients. **b** Comparison of scores of BER, NER and HRR pathways between patients with or without ATRX mutation. **c** ATRX mutant female patients showed higher scores of BER, NER and HRR pathways than patients without ATRX mutation. **d** The expression of core genes in three DDR related pathways between four subgroups. Sub1: female patients with ATRX mutation; Sub2: female patients without ATRX mutation; Sub3: male patients with ATRX mutation; Sub4: male patients without ATRX mutation

We performed GSEA to analyze the functional context of ATRX mutation. KEGG pathway analysis showed ATRX mutation was mainly enriched in base excision repair (BER), nucleotide excision repair (NER) and homologous recombination repair (HRR) (Fig. 6a). The biological function of ATRX mutation was significantly involved in DNA damage repair (DDR). We then used the ssGSEA to characterize the pathway of BER, NER and HRR between ATRX mutant and wild patients. As illustrated in Fig. 6b, BER (median score 0.8734 vs 0.7909, *P* = 0.0002), NER (median score 0.4333 vs 0.3605, *P* = 0.044) and HRR (median score 0.7618 vs 0.6898, *P* = 0.0098) were significantly enriched in ATRX mutant patients. Furthermore, we continued to explore the effect of ATRX mutation on BER, NER and HRR pathways stratified by sex. Only female patients with ATRX mutation manifested higher scores of BER, NER and HRR than other patients without ATRX mutation **(**Fig. 6c**)**.

Moreover, we chose the core genes enriched in these three pathways to examine the expression differences between ATRX mutant and wild patients. Interestingly, most core genes expressed highly in ATRX mutant type patients when comparing that of wild type patients (Fig. 6d). After sex stratification, the expression levels of these DDR-related core genes were also higher in subgroups of female and male patients with ATRX mutation than other two wild type subgroups (Additional file 6).

Taken together, these results implied the possibly enhanced activation of DDR pathways in ATRX mutant patients. The higher TMB and activation of DDR system might indicate the likelihood of fierce mutagenesis and corresponding compensatory DDR activation in ATRX mutant patients.

### ATRX mutation is associated with favorable clinical benefit to ICI

**Fig. 7:**
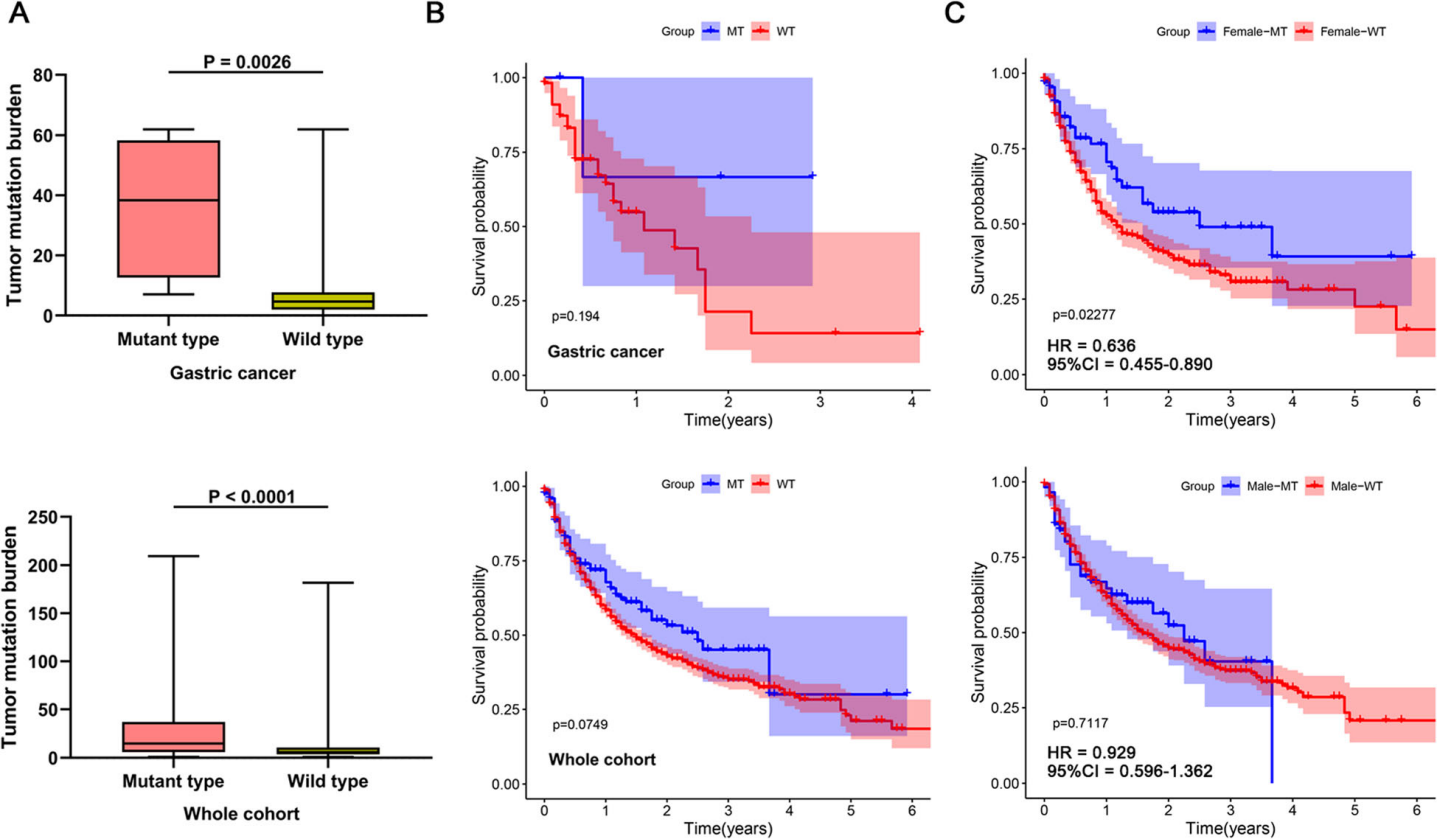
ATRX mutation is associated with favorable clinical benefit to ICI. **a** Comparison of TMB between ATRX mutant and wild type ICI-treated patients in sub cohort of gastric cancer and the whole cohort. **b** Kaplan–Meier analysis of comparing overall survival between patients with or without ATRX mutation in sub cohort of gastric cancer and the whole cohort. **c** Correlation of ATRX mutation with overall survival benefits stratified by sex in the whole cohort. Female patients with ATRX mutation obtained significantly prolonged overall survival compared with wild type female patients. The HR of ATRX mutant type vs wild type for female patients (HR = 0.636, 95%CI = 0.455–0.890, *P* = 0.023) is better than the HR for male patients (HR = 0.929, 95%CI = 0.596–1.362, *P* = 0.7117)

In order to investigate whether patients with ATRX mutation could benefit from ICI, we obtained the publicly available genomic and survival data of MSKCC ICI-treated cohort from cbioportal database [15]. The MSKCC ICI-treated cohort contains 1610 patients of various cancer types with mutation data including 54 gastric cancer patients. The mutation frequency of ATRX in the gastric or gastroesophageal junction cancer patients is 7.41% (4/54). We observed that patients with ATRX mutation had higher TMB than ATRX wild type patients (Fig. 7a). In the patients of gastric cancer, ATRX mutation patients trended toward a longer overall survival (median, not reached vs 13 months, log rank *P* = 0.194) (Fig. 7b). Similarly, the overall survival of patients with ATRX mutation in the whole cohort was also superior to that of ATRX wild patients (median, 30 months vs 18 months, log rank *P* = 0.075) (Fig. 7b). We then divided the whole cohort into two groups (female and male) and compared the overall survival between ATRX mutation status. Interestingly, only female patients with ATRX mutation obtained significantly prolonged overall survival compared with wild type female patients (median, 30 months vs 14 months, log rank *P* = 0.023) (Fig. 7c). Furthermore, we compared the prediction power of ATRX mutation in male and female patients. The HR of ATRX mutant type vs wild type for female patients (HR = 0.636, 95%CI = 0.455–0.890, *P* = 0.023) is better than the HR for male patients (HR = 0.929, 95%CI = 0.596–1.362, *P* = 0.7117) (Fig. 7c).

Collectively, these data suggested that ATRX mutation especially in female patients was associated with favorable clinical benefit to ICI treatment.

## Discussion

In this study, through comparing somatic mutation profiles derived from male and female GC patients, we identified for the first time that the ATRX mutation preferentially occurred in female GC patients was associated with higher TMB, increased anticancer immunity and favorable clinical benefit to ICI.

ATRX is a tumor suppressor gene and encodes a member of the SWI/SNF family of proteins. ATRX was demonstrated to be associated with DNA damage repair [29], maintaining genetic stability [30], facilitating appropriate DNA replication [31] and affecting the expression of specific genes [32]. ATRX mutation constitutes the common genetic abnormalities in gliomas. In IDH-wild type gliomas, the presence of ATRX mutations served as a favorable marker of longer patient survival [33]. The loss of ATRX impaired the non-homologous end joining activity and increased MSI in glioblastoma, rendering higher sensitivity to DNA-damaging agents [34]. Han et al. reported that ATRX could regulate DNA damage repair through modulating the ATM pathway and increase the sensitivity to temozolomide in glioma [35]. In our study, higher TMB and activation of DNA damage repair pathways were observed in female patients with ATRX mutation. TMB results from the confrontation between mutation and DNA repair. These results suggested that the function of compensatory DNA damage repair might be weaker than fiercer mutagenesis with ATRX mutation. We have speculated that the ATRX mutation in female GC patients could impact the related DNA damage repair, accounting for the higher TMB and corresponding enhanced anticancer immunity. Nevertheless, the precise mechanism of ATRX mutation in regulating anticancer immunity in female GC patients requires further research.

Existing researches have reported the sex biases of ATRX. Young et al. have discovered that female mice lacking ATRX in pancreas exclusively showed increased sensitivity to injury and the oncogenic action of mutated KRAS, whereas male mice without ATRX were protected [36]. Similarly, we also found that female patients with ATRX mutation showed enhanced immunogenicity, especially stronger anticancer immunity than ATRX mutant male patients, which might be partly due to increased sensitivity to DNA damage in ATRX mutant female patients. Dunford et al. have reported that a fraction of tumor suppressor genes (including ATRX) in chrX might escape inactivation during embryogenesis, leading to biallelic expression of these genes and enhanced cancer protection in female [37]. This research might explain the result that no significant decrease of ATRX expression in female GC patients with ATRX mutation, although truncating and deleterious missense mutations were predominant in ATRX mutant female patients.

In addition to the sex differences in immunogenicity between male and female patients, sex disparities also existed in the performance of ATRX mutation in ICI prediction. Although longer overall survival was observed in patients with ATRX mutation treated with ICI, the predictive power of ATRX mutation was better for female than male patients. Female patients with ATRX mutation acquired significantly greater survival benefits from ICI treatment. Wang et al. have also reported a similar result that TMB showed better predictive power in ICI response for female NSCLC patients than male patients [38]. Future development and application of ICI biomarkers should take sex differences into account.

There are several limitations in this study. First, the overall mutation frequency of ATRX in GC patients is relatively low, in particular for male GC patients. Moreover, we only obtained the transcriptome data from a single cohort. These limitations could influence the strength of the comparison of anticancer immunity between female and male patients. Second, the ATRX mutation frequency varies in different populations. In our study, the ATRX mutation frequency in three GC cohorts was different (31.7% in GACA-CN cohort, 6.3% in GACA-JP cohort and 5.3% in TCGA cohort). Furthermore, the proportions of female patients with ATRX mutation in GACA-CN (40.5%) were much higher than other two cohorts (12.3% in TCGA cohort and 11.0% in GACA-JP cohort). The difference of ATRX mutation frequency in different populations might somehow limit the use of ATRX mutation as a favorable prognosis biomarker. Third, our analysis only proved the association of ATRX mutation with TMB, anticancer immunity and the efficacy of ICI therapy. The potential mechanism of ATRX mutation in ICI treatment especially for male patients still needs further exploration. Finally, it would be better to compare the survival benefits from ATRX mutation between female and male GC patients after ICIs treatment, which could directly validate the sex differences of ATRX mutation in immunotherapy-related anticancer immunity. However, we failed to make the comparison due to the limited sample size of ATRX mutant GC patients in the MSKCC ICI-treated cohort. Therefore, we tried to preliminarily explore whether might be sex differences in survival benefits from ATRX mutation through comparing the differences of prognosis outcomes from ATRX mutation between female and male patients across various cancer types in the whole cohort. Although greater survival benefits from ICIs treatment were observed in female patients with ATRX mutation across various cancer types, these data may over exaggerate the conclusion drawn for GC patients. More ICI-treated cohorts containing larger sample size of ATRX mutant GC patients are required to validate the sex differences in survival benefits herein.

## Conclusions

In summary, our study found that ATRX preferentially mutating in female GC patients was associated with the enhanced immunogenicity, increased anticancer immunity and favorable clinical benefit to ICI. The sex disparities in mutation frequency, anticancer immunity and clinical benefits suggested that ATRX mutation might be more appropriate as a potential predictor for favorable clinical benefit to ICI in female GC patients. ATRX mutation could be combined with other biomarkers of ICI response to better identify the female GC patients who will derive greater benefits from immunotherapy. However, further clinical researches are required to validate our results and to assess the value of ATRX mutation in male patients.

## Supplementary Information

**Additional file 1.** The gene list of ssGSEA

**Additional file 2.** The differences of ATRX mutation frequency between female and male tumor patients

**Additional file 3.** The impact of ATRX missense mutation on protein structure

**Additional file 4.** Comparison of overall survival probability between patients with or without ATRX mutation in non-GC cohorts with ATRX mutation frequency over 10% from TCGA database.

**Additional file 5.** Comparison of the TMB and anticancer immunity scores between female and male GC patients. a. Boxplot showing the TMB and anticancer immunity scores between female and male GC patients. Only TMB showed the significantly differences between female and male. b. The differences of TMB between female and male GC patients disappeared when excluding the TMB of ATRX mutant female patients.

**Additional file 6.** Comparison of the expression of DDR-related core genes between four subgroups. Sub1: female patients with ATRX mutation; Sub2: female patients without ATRX mutation; Sub3: male patients with ATRX mutation; Sub4: male patients without ATRX mutation

## Data Availability

All the data used and analyzed in this study are available in the TCGA (https://portal.gdc.cancer.gov/), UCSC Xena (http://xena.ucsc.edu/) and cbioportal repository (https://www.cbioportal.org/). Public access to the data of this study is open.

## Abbreviations

GC: Gastric cancer
ICI: Immune checkpoint inhibitors
TCGA: The cancer genome atlas
ICGC: International Cancer Genome Consortium
FDR: False discovery rate
TMB: Tumor mutation burden
CYT: Cytolytic activity
APM: Antigen presenting machinery
GSEA: Gene set enrichment analysis
ssGSEA: Single sample gene set enrichment analysis
MSI: Microsatellite instability
BER: Base excision repair
NER: Nucleotide excision repair
HRR: Homologous recombination repair
DDR: DNA damage repair
