# Supplementary material for: The association of sex-biased ATRX mutation in female gastric cancer patients with enhanced immunotherapy-related anticancer immunity
Source: BMC Cancer. 2021 Mar 7;21:240. doi: 10.1186/s12885-021-07978-3 (PMC7938533; doi:10.1186/s12885-021-07978-3)
Supplement: Supplementary file 1 — Additional file 1. The gene list of ssGSEA [file 12885_2021_7978_MOESM1_ESM.docx]

**Additional file 1. The gene list of ssGSEA**

|  | APM | KEYNOTE012 | KEYNOTE059 | BER | NER | HRR |
| --- | --- | --- | --- | --- | --- | --- |
| Gene  list | HLA-A | CXCL9 | CCL5 | APEX1 | CDK7 | BLM |
|  | HLA-B | CXCL10 | CD27 | APEX2 | CETN2 | BRCA2 |
|  | HLA-C | IDO1 | CD274 | FEN1 | CUL4A | EME1 |
|  | B2M | IFNG | CD276 | HMGB1 | CUL4B | MRE11 |
|  | TAP1 | HLA-DRA | CD8A | HMGB1P1 | DDB1 | MUS81 |
|  | TAP2 | STAT1 | CMKLR1 | HMGB1P40 | DDB2 | NBN |
|  | TAPBP |  | CXCL9 | LIG1 | ERCC1 | POLD1 |
|  |  |  | CXCR6 | LIG3 | ERCC2 | POLD2 |
|  |  |  | HLA-DQA1 | MBD4 | ERCC3 | POLD3 |
|  |  |  | HLA-DRB1 | MPG | ERCC4 | POLD4 |
|  |  |  | HLA-E | MUTYH | ERCC5 | RAD50 |
|  |  |  | IDO1 | NEIL1 | ERCC6 | RAD51 |
|  |  |  | LAG3 | NEIL2 | ERCC8 | RAD51B |
|  |  |  | NKG7 | NEIL3 | GTF2H1 | RAD51C |
|  |  |  | PDCD1LG2 | NTHL1 | GTF2H2 | RAD51D |
|  |  |  | PSMB10 | OGG1 | GTF2H3 | RAD52 |
|  |  |  | STAT1 | PARP1 | GTF2H4 | RAD54B |
|  |  |  |  | PARP2 | GTF2H5 | RAD54L |
|  |  |  |  | PARP3 | LIG1 | RPA1 |
|  |  |  |  | PARP4 | MNAT1 | RPA2 |
|  |  |  |  | PCNA | PCNA | RPA3 |
|  |  |  |  | POLB | POLD1 | RPA4 |
|  |  |  |  | POLD1 | POLD2 | SEM1 |
|  |  |  |  | POLD2 | POLD3 | SSBP1 |
|  |  |  |  | POLD3 | POLD4 | TOP3A |
|  |  |  |  | POLD4 | POLE | TOP3B |
|  |  |  |  | POLE | POLE2 | XRCC2 |
|  |  |  |  | POLE2 | POLE3 | XRCC3 |
|  |  |  |  | POLE3 | POLE4 |  |
|  |  |  |  | POLE4 | RAD23A |  |
|  |  |  |  | POLL | RAD23B |  |
|  |  |  |  | SMUG1 | RBX1 |  |
|  |  |  |  | TDG | RFC1 |  |
|  |  |  |  | UNG | RFC2 |  |
|  |  |  |  |  | RFC3 |  |
|  |  |  |  |  | RFC4 |  |
|  |  |  |  |  | RFC5 |  |
|  |  |  |  |  | RPA1 |  |
|  |  |  |  |  | RPA2 |  |
|  |  |  |  |  | RPA3 |  |
|  |  |  |  |  | RPA4 |  |
|  |  |  |  |  | XPA/XPC |  |
