# Supplementary material for: The association of sex-biased ATRX mutation in female gastric cancer patients with enhanced immunotherapy-related anticancer immunity
Source: BMC Cancer. 2021 Mar 7;21:240. doi: 10.1186/s12885-021-07978-3 (PMC7938533; doi:10.1186/s12885-021-07978-3)
Supplement: Supplementary file 2 — Additional file 2. The differences of ATRX mutation frequency between female and male tumor patients [file 12885_2021_7978_MOESM2_ESM.docx]

**Additional file 2. The differences of ATRX mutation frequency between female and male tumor patients**

| Project | Mutation frequency | Sex | MT | WT | Fisher exact P value |
| --- | --- | --- | --- | --- | --- |
| BTCA-SG | 81.7% | Female | 29 (96.7%) | 1 (3.3%) | **0.005** |
|  |  | Male | 29 (70.7%) | 12 (29.3%) |  |
| LMS-FR | 74.4% | Female | 42 (79.2%) | 9 (20.8%) | 0.091 |
|  |  | Male | 8 (57.1%) | 6 (42.9%) |  |
| MELA-AU | 73.2% | Female | 60(76.9%) | 18 (23.1%) | 0.401 |
|  |  | Male | 74 (71.1%) | 30 (28.9%) |  |
| LIRI-JP | 55.0% | Female | 47 (70.1%) | 20 (29.9%) | **0.004** |
|  |  | Male | 95 (49.7%) | 96 (50.3%) |  |
| SKCA-BR | 49.0% | Female | 23 (54.7%) | 19 (45.3%) | 0.418 |
|  |  | Male | 26 (44.8%) | 32 (55.2%) |  |
| MALY-DE | 45.6% | Female | 74 (64.3%) | 41 (35.7%) | **<0.001** |
|  |  | Male | 36 (28.5%) | 90 (71.5%) |  |
| PAEN-IT | 35.1% | Female | 6 (46.1%) | 7 (53.9%) | 0.472 |
|  |  | Male | 7 (29.1%) | 17 (70.9%) |  |
| PACA-CA | 32.9% | Female | 61 (53.0%) | 54 (47.0%) | **< 0.001** |
|  |  | Male | 23 (15.8%) | 123 (84.2%) |  |
| LGG-US | 23.4% | Female | 57 (25.1%) | 170 (74.9%) | 0.461 |
|  |  | Male | 62 (22.1%) | 219 (77.9%) |  |
| LICA-CN | 21.4% | Female | 22 (18.9%) | 41 (81.1%) | **0.007** |
|  |  | Male | 64 (57.1%) | 274 (57.1%) |  |
| LUSC-KR | 20.6% | Female | 4 (10.5%) | 34 (89.5%) | 0.110 |
|  |  | Male | 31 (23.5%) | 101 (76.5%) |  |
| THCA-CN | 20.0% | Female | 4 (12.1%) | 29 (87.9%) | 0.070 |
|  |  | Male | 6 (35.3%) | 11 (64.7%) |  |
| PBCA-US | 17.7% | Female | 24 (28.2%) | 61 (71.8%) | **0.001** |
|  |  | Male | 9 (8.9%) | 92 (71.8%) |  |
| PBCA-DE | 16.0% | Female | 48 (28.2%) | 175 (71.8%) | **0.003** |
|  |  | Male | 32 (11.6%) | 244 (88.4%) |  |
| PACA-AU | 15.9% | Female | 42 (23.5%) | 137 (76.5%) | **< 0.001** |
|  |  | Male | 20 (9.0%) | 201 (91.0%) |  |
| PAEN-AU | 15.4% | Female | 4 (21.1%) | 15 (78.9%) | 0.443 |
|  |  | Male | 4 (12.1%) | 29 (87.9%) |  |
| BOCA-UK | 12.3% | Female | 11 (23.9%) | 35 (76.1%) | **0.004** |
|  |  | Male | 5 (6.2%) | 76 (93.8%) |  |
| LICA-FR | 11.1% | Female | 11 (20.4% ) | 43 (79.6%%) | **0.015** |
|  |  | Male | 17 (8.6%%) | 224 (91.4 %) |  |
| SARC-US | 10.4% | Female | 15 (11.5%) | 115 (88.5%) | 0.672 |
|  |  | Male | 10 (9.1%) | 100 (90.9%) |  |
| SKCM-US | 10.3% | Female | 25 (21.4%) | 92 (78.6%) | **< 0.001** |
|  |  | Male | 23 (8.0%) | 266 (92.0%) |  |
| COCA-CN | 10.3% | Female | 15 (11.8%) | 112 (88.2%) | 0.460 |
|  |  | Male | 18 (9.3%) | 176 (90.7%) |  |
| PEME-CA | 9.9% | Female | 4 (12.5%) | 28 (87.5%) | 0.721 |
|  |  | Male | 6 (8.7%) | 63 (91.3 %) |  |
| LUAD-US | 8.5% | Female | 34 (12.3%) | 243 (87.7%) | **0.001** |
|  |  | Male | 10 (4.2%) | 229 (95.8%) |  |
| ORCA-IN | 8.4% | Female | 1 (3.8%) | 25 (96.2%) | 0.701 |
|  |  | Male | 14 (9.2 %) | 138 (90.8%) |  |
| COAD-US | 7.9% | Female | 24 (12.5%) | 168 (87.5%) | **0.001** |
|  |  | Male | 8 (3.8%) | 202 (96.2%) |  |
| RECA-EU | 7.8% | Female | 17 (9.6%) | 160 (90.4%) | 0.273 |
|  |  | Male | 16 (6.5%) | 229 (93.5%) |  |
| CLLE-ES | 7.7% | Female | 24 (11.7%) | 182 (88.3%) | **0.006** |
|  |  | Male | 15 (4.9%) | 289 (95.1 %) |  |
| LAML-KR | 7.3% | Female | 9 (11.5%) | 69 (88.5%) | 0.417 |
|  |  | Male | 6 (6.9%) | 81 (93.1%) |  |
| GBM-US | 7.2% | Female | 13 (9.2%) | 129 (90.8%) | 0.309 |
|  |  | Male | 15 (6.1%) | 231 (93.9%) |  |
| LINC-JP | 6.4% | Female | 11 (11.6%) | 84 (88.4%) | **0.027** |
|  |  | Male | 14 (4.7%) | 285 (95.3%) |  |
| READ-US | 6.3% | Female | 4 (6.1%) | 62 (93.9%) | 1 |
|  |  | Male | 5 (6.5%) | 72 (93.5%) |  |
| LUSC-US | 5.8% | Female | 13 (10.2%) | 115 (89.8%) | **0.025** |
|  |  | Male | 15 (4.2%) | 342 (95.8%) |  |
| BLCA-US | 5.6% | Female | 8 (7.5%) | 99 (92.5%) | 0.333 |
|  |  | Male | 15 (4.9%) | 289 (95.1%) |  |
| HNSC-US | 5.3% | Female | 15 (10.9%) | 123 (89.1%) | **0.001** |
|  |  | Male | 12 (3.2%) | 358 (96.8%) |  |
