# Supplementary material for: The association of sex-biased ATRX mutation in female gastric cancer patients with enhanced immunotherapy-related anticancer immunity
Source: BMC Cancer. 2021 Mar 7;21:240. doi: 10.1186/s12885-021-07978-3 (PMC7938533; doi:10.1186/s12885-021-07978-3)
Supplement: Supplementary file 3 — Additional file 3. The impact of ATRX missense mutation on protein structure [file 12885_2021_7978_MOESM3_ESM.docx]

**Additional file 3. The impact of ATRX missense mutation on protein structure**

| Gene symbol | Tumor sample barcode | Sex | Consequence | PolyPhen-2 score |
| --- | --- | --- | --- | --- |
| ATRX | TCGA-D7-6528 | Female | Missense variant | 0.995 |
| ATRX | TCGA-D7-A4YV | Female | Missense variant | 0.911 |
| ATRX | TCGA-BR-8366 | Female | Missense variant | 0.988 |
| ATRX | TCGA-BR-8361 | Female | Missense variant | 0.977 |
| ATRX | TCGA-BR-7704 | Female | Missense variant | 0.969 |
| ATRX | TCGA-CD-8524 | Female | Missense variant | 0.921 |
| ATRX | TCGA-BR-7707 | Female | Missense variant | 0.879 |
| ATRX | TCGA-SW-A7EA | Female | Missense variant | 0.874 |
| ATRX | TCGA-CD-8531 | Female | Missense variant | 0.696 |
| ATRX | TCGA-BR-8363 | Female | Missense variant | 0.272 |
| ATRX | TCGA-D7-6528 | Female | Missense variant | 0.022 |
| ATRX | TCGA-BR-4368 | Female | Missense variant | 0.014 |
| ATRX | TCGA-BR-6852 | Female | Missense variant | 0.007 |
| ATRX | TCGA-D7-A6EX | Female | Splice donor variant | - |
| ATRX | TCGA-HU-A4GT | Female | Frameshift variant | - |
| ATRX | TCGA-HU-A4H4 | Female | Frameshift variant | - |
| ATRX | TCGA-BR-6452 | Female | Frameshift variant | - |
| ATRX | TCGA-HU-A4G8 | Female | Frameshift variant | - |
| ATRX | TCGA-MX-A5UJ | Female | Frameshift variant | - |
| ATRX | TCGA-BR-6452 | Female | 3 prime UTR variant | - |
| ATRX | TCGA-BR-4363 | Female | 3 prime UTR variant | - |
| ATRX | TCGA-BR-8680 | Male | Missense variant | 0.109 |
| ATRX | TCGA-VQ-A8PC | Male | Missense variant | 0.014 |
| ATRX | TCGA-CD-8535 | Male | Frameshift variant | - |
| ATRX | TCGA-D7-5578 | Male | Synonymous variant | - |
