# Supplementary material for: The association of sex-biased ATRX mutation in female gastric cancer patients with enhanced immunotherapy-related anticancer immunity
Source: BMC Cancer. 2021 Mar 7;21:240. doi: 10.1186/s12885-021-07978-3 (PMC7938533; doi:10.1186/s12885-021-07978-3)
Supplement: Supplementary file 4 — Additional file 4. Comparison of overall survival probability between patients with or without ATRX mutation in non-GC cohorts with ATRX mutation frequency over 10% from TCGA database. [file 12885_2021_7978_MOESM4_ESM.pdf]

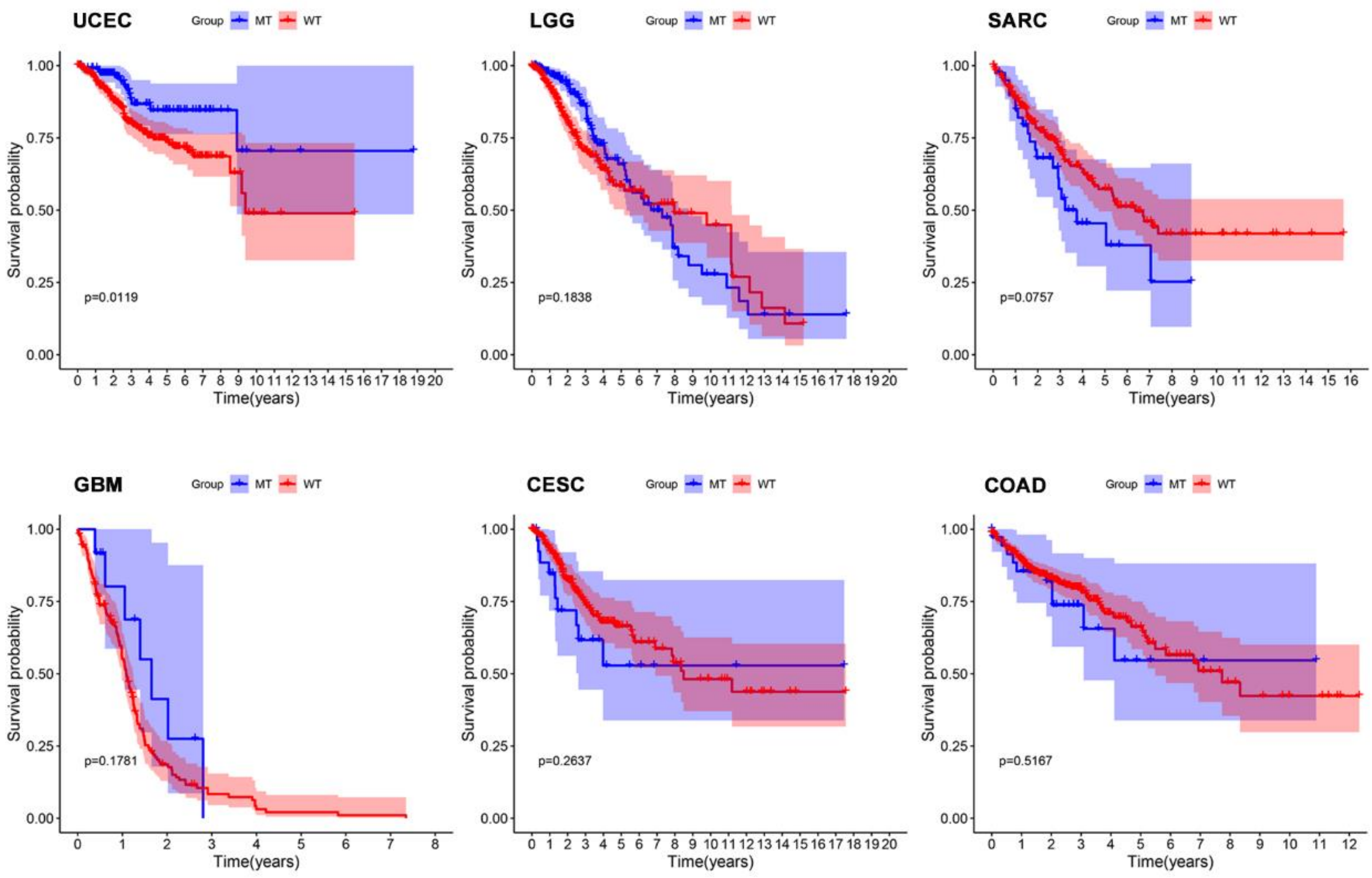

Additional file 4. Comparison of overall survival probability between patients with or without ATRX mutation in non-GC cohorts with ATRX mutation frequency over 10% from TCGA database.
