# Supplementary material for: The association of sex-biased ATRX mutation in female gastric cancer patients with enhanced immunotherapy-related anticancer immunity
Source: BMC Cancer. 2021 Mar 7;21:240. doi: 10.1186/s12885-021-07978-3 (PMC7938533; doi:10.1186/s12885-021-07978-3)
Supplement: Supplementary file 5 — Additional file 5. Comparison of the TMB and anticancer immunity scores between female and male GC patients. a. Boxplot showing the TMB and anticancer immunity scores between female and male GC patients. Only TMB showed the significantly differences between female and male. b. The differences of TMB between female and male GC patients disappeared when excluding the TMB of ATRX mutant female patients. [file 12885_2021_7978_MOESM5_ESM.pdf]

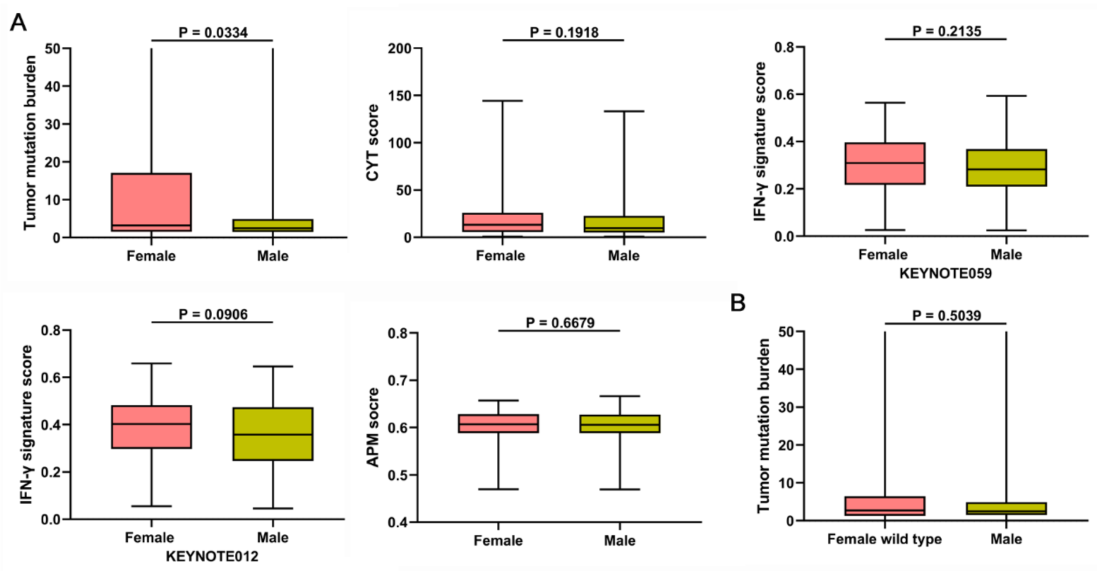

Additional file 5. Comparison of the TMB and anticancer immunity scores between female and male GC patients. **a.** Boxplot showing the TMB and anticancer immunity scores between female and male GC patients. Only TMB showed the significantly differences between female and male. **b.** The differences of TMB between female and male GC patients disappeared when excluding the TMB of ATRX mutant female patients.
