# Supplementary material for: The association of sex-biased ATRX mutation in female gastric cancer patients with enhanced immunotherapy-related anticancer immunity
Source: BMC Cancer. 2021 Mar 7;21:240. doi: 10.1186/s12885-021-07978-3 (PMC7938533; doi:10.1186/s12885-021-07978-3)
Supplement: Supplementary file 6 — Additional file 6. Comparison of the expression of DDR-related core genes between four subgroups. Sub1: female patients with ATRX mutation; Sub2: female patients without ATRX mutation; Sub3: male patients with ATRX mutation; Sub4: male patients without ATRX mutation [file 12885_2021_7978_MOESM6_ESM.pdf]

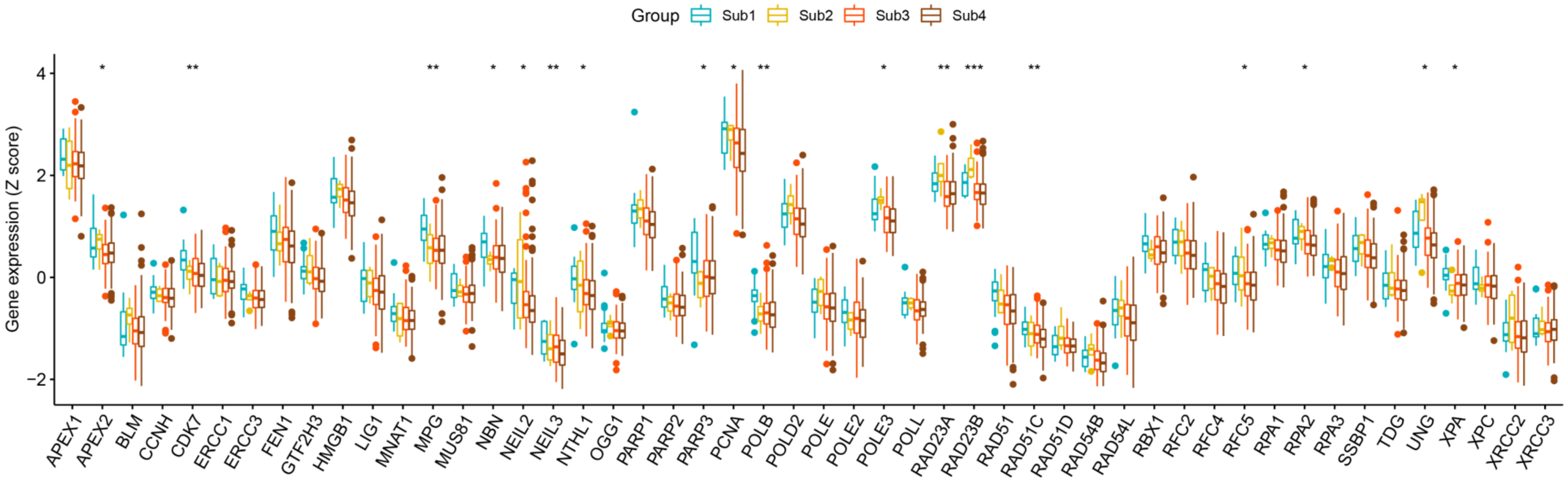

Additional file 6. Comparison of the expression of DDR-related core genes between four subgroups. Sub1: female patients with ATRX mutation; Sub2: female patients without ATRX mutation; Sub3: male patients with ATRX mutation; Sub4: male patients without ATRX mutation
